# Why Theory of Mind Is Not Enough to Understand Others?

**DOI:** 10.3390/bs13010012

**Published:** 2022-12-23

**Authors:** María Isabel Sanhueza, Pablo Fossa

**Affiliations:** Facultad de Psicología, Universidad del Desarrollo, Santiago 7610658, Chile

**Keywords:** theory of mind, reflective consciousness, pre-reflective consciousness, intuition, phenomenology

## Abstract

Theory of Mind (ToM), understood as the ability to intuit one’s own mental states and those of others, has been extensively researched in developmental psychology and cognitive psychology. The psychological literature shows a direct relationship between ToM and the (self) reflective capacity of consciousness, a product of the cognitive effort that implies the understanding of one’s own subjectivity and that of others. In this sense, ToM has received a strong cognitive influence, sub-dimensioning other dimensions involved in the intersubjective process of mutual understanding. Based on the theory of pre-reflective consciousness and the theory of intuition in phenomenology, we propose in this paper that the process of understanding one’s own mental states and the mental states of others constitutes, mainly, a pre-reflective and intuitive experience, and that it is only possible to move on to reflection at a later time. In short, with contributions from the theory of pre-reflective consciousness and phenomenological intuition, the aim is to complement the theoretical bases of ToM in psychology; a theory that, without incorporating elements of phenomenology, remains incomplete.

## 1. Introduction

The notion of Theory of Mind (ToM) in psychology has been understood as the ability to understand and predict one's own behaviour and that of others, accounting for their knowledge, emotions, intentions and beliefs [1]. ToM has also been related to the phenomenon of mentalization, since it alludes to a metacognitive ability that allows elucidating how a cognitive system manages to notice the events of a different cognitive system. That is, ToM constitutes a complex cognitive ability through which an individual manages to attribute mental states to himself and others in order to interpret, explain and predict the internal life or actions of himself and others [1,2,3].

ToM has been a relevant study phenomenon in the development of psychology during the 20th century and has had important repercussions for developmental psychology and cognitive psychology. This phenomenon has shed light on how the human encounter and the intersubjective process occur, allowing us to decipher the complex psychological framework that implies what has been a focus of interest for much of the history of psychology: the bond with another. The influence of ToM in mentalization theory, attachment theory, couple relationships, teaching-learning processes and psychotherapy is diverse. In general, all of its theoretical development allows us to get closer to understanding how the bond with others is co-constructed and developed, and how mutual understanding is produced in our daily interactions. ToM constitutes one of the processes of the self. For example, ToM has been related to phenomena such as emotional regulation, self-regulation and self-reflection. In this sense, the literature has described that the development of a theory about oneself and about others implies second-order reflexive processes, in which all the executive functions would be at the service of a controlled and directed cognitive activity as an introspective process focused on the analysis of the subjective experience of the other. The phenomenon of introspection, for its part, so widely studied in the history of psychology [4,5] takes a central place here. Introspection, self-reflection and ToM seem to be intertwined in the process of understanding oneself and others. Reflexivity, in particular, takes a special place in all theoretical descriptions of the notion of ToM [1,2,3]. The reflective dimension of consciousness sets in motion the cognitive mechanisms, executive functions and control of thought necessary and fundamental to carry out an introspective process to know oneself and the analytical-reflexive process to develop a theory about the subjectivity of others. Phenomenology, for its part, a branch of philosophy that has focused its efforts on the study of transcendental subjectivity, has made important contributions to theoretical and empirical research in psychology. However, these contributions have been under-dimensioned due to the criticisms that phenomenology delivers to empirically generated knowledge. Mainstream research in psychology has resisted a phenomenological understanding of human experience because of phenomenology’s explicit critiques of psychologism [6,7,8,9].

One of the fundamental notions of phenomenological philosophy consists of pre-reflective consciousness and the theory of intuition. From phenomenological approaches, there is a pre-reflective dimension of consciousness, a first-order, non-thematic, non-theoretical functioning, which differs from the empirical self of psychological consciousness. This dimension is a basal functioning of consciousness, passive, that is, not controlled or reflective [6,7,10,11,12]. This pre-reflective dimension of consciousness is not exclusive to the reflective dimension, but both dimensions are present in the flow of life and in the encounter with the objects of the world. The pre-reflective dimension generates the bases for a reflexive consciousness. The pre-reflective consciousness is a lived and felt consciousness, not thought, not thematized. This dimension of consciousness lays the foundations for reflexivity since it is only possible to re-flect (return to something or return to) when there is a previous lived experience. This pre-reflective dimension of consciousness is directly linked to the theory of intuition. From phenomenological philosophy, knowledge of oneself and of the world is produced via intuition and not through reason (reflection) [7,9]. Intuition is the direct and immediate perception of the objects of the world, which is experienced in a pre-reflective way, and it is only possible to reflect at a later time. Pre-reflective consciousness receives intuitively perceived objects through direct experience of essences [8]. In the pre-reflective consciousness, the objects of immediate perception are grasped directly, abruptly and intuitively, and constitute the purest phenomenological essence of the objects in the world.

Phenomenological intuition and pre-reflective consciousness constitute psychological phenomena that underlie reflection and ToM. This article aims to defend the idea that ToM in psychology is not enough to understand one’s own subjective states and those of others, but rather requires a pre-reflective and intuitive intersubjective approach. Only with contributions from the theory of intuition and pre-reflective consciousness is it possible to complete ToM in psychology and fully understand the process of mutual understanding during human interactions.

## 2. Theory of Mind (ToM) in Psychology

For some cognitivist currents, the brain could work as a predictive machine that seeks to reduce the uncertainty of the environment with an adaptive purpose. For this, the executive functions facilitate the ability to generate solutions in the face of a problematic stimulus by predicting the measures of the possible consequences of the visualized solutions. In this way, a better adaptation to the context and an efficient approach to adversity are achieved. However, the human being also generates predictions about the behaviours, thoughts, beliefs, emotions and intentions of others, produced by ToM [13].

The first studies in psychology on the so-called ToM date back to 1971 when Dennett proposed the notion of an intentional attitude as a way through which people give meaning to the behaviour of others. Subsequently, Premack and Woodruff, establishing a direct relationship between Theory of Mind and Mentalization, propose that an individual has the ability to mentalize and attribute mental states to himself and to other people [14]. In this way, ToM facilitates the emergence of inferences before states that are not directly observable and the prediction of subsequent behaviours. The insertion of this phenomenon in developmental psychology arose with the works published by Wimmer and Perner in 1983, where the ability of children between 4 and 5 years of age to understand false beliefs was estimated. During the year 1985, Baron Cohen, Leslie and Frith carried out a study based on the existence of ToM in children with autism, where the empirical data showed the lack of this ability. Over time, studies and research increased exponentially, giving rise to a large-scale phenomenon within psychology [14].

The mind and consciousness correspond to highly complex entities, since they involve not only brain interaction but also the social context where the processes emerge and turn their functionality into the development of processes and dynamics that allow the adaptation of the human being to the social and cultural world. Different theoretical positions and disciplines have argued that the human being is in a constant effort to understand and explain himself in relation to the mind and social cognition. The human being corresponds to a mostly social species, since he develops in a context of interactions through a wide range of cognitions related to identity, interactions and roles. Social cognition maintains its basis in the interpretation of oneself and others in terms of internal mental experiences. For this, ToM as a theoretical construct has contributed to several significant contributions to the understanding of the mind, cognitive and socio-cultural phenomena, being a subject in growing development [1,2,3].

The concept of ToM is delimited as the ability to understand and predict one's own behaviour and that of others, accounting for their knowledge, emotions, intentions and beliefs [1,3,13]. This phenomenon, often also linked to the concept of mentalization, alludes to a metacognitive ability by elucidating how a cognitive system manages to notice the events of a different cognitive system. In this way, it is constituted as a complex cognitive ability through which an individual attributes mental states of himself and others in order to interpret, explain and predict the internal life or actions of himself and others. Through prediction, it is sought that each subject can share and function in various interpersonal contexts, achieving their goals, developing skills and facing challenges. ToM presents various levels of complexity, referring to facial recognition, first- and second-order beliefs, social use of language, social behaviour and empathy, among others [1,3,13].

Both philosophers and psychologists share that the reasoning of ToM is structured in broad categorizations linked to the mind and behaviour that must include constructs related to affect and perception to achieve a deep approach. From this, people's perceptions and emotions provide a basis for beliefs and desires. For their part, beliefs and desires guide actions and shape the reactions of the environment in the face of a specific act [2].

ToM has been the subject of a consistent research effort and has become an important theoretical construct that integrates various contributions. From the perspective of Piaget’s [15] cognitive development, four successive stages of cognitive development are structured within the evolutionary cycle that account for the cognitive abilities and capacities that children naturally develop at different stages. ToM begins to develop itself between 3 and 7 years of age, during the preoperational stage, where the child acquires skills from a basic level to a more complex one. In the first instance, they can detect the desire of other people, make use of symbolic play and understand first-order false beliefs to later develop the inference of emotional states according to a social context and the ability to read, interpret and predict mental states [16,17].

The neurological bases that explain this central phenomenon of social cognition are related to various brain regions, mainly the prefrontal cortex, temporal–parietal conjunction, medial parietal cortex and amygdala [13,18].

At all stages of the life cycle, ToM is a crucial sociocognitive understanding. However, in early stages it directly influences the school experience, academic results and interpersonal relationships, particularly linked to popularity among peers, acceptance, teacher–student interaction and adoption of leadership roles. Likewise, it favours better levels of performance and learning in relation to mathematical abilities, communication strategies and understanding of instructions [2]. In short, ToM has been understood in psychology as an achievement of cognitive development and an expression of social cognition. ToM as a psychological function plays a fundamental role in social communication, empathy, and mutual understanding during human interactions, and has been understood as a dimension of reflective consciousness in human experience. However, it lacks an understanding of how pre-reflective elements of consciousness may be involved in the theory we build about ourselves and others.

## 3. Pre-Reflective Dimension of Consciousness

Phenomenology does not conceive consciousness from reflection or metacognitive processes, but rather arises from a primary experience. In this way, it postulates various levels of consciousness that account for the modes of meaning and relationship that the subject has in interaction with the world. In the first instance, there is the pre-reflective consciousness, characterized by a passive dimension and, later, the reflective consciousness, which maintains an active and intentional character [10,19,20].

Reflection corresponds to a higher process that allows self-awareness as an object; that is, the fact of being able to refer to and think of oneself in the same way that one can refer to the objects of the world. Thus, the “reflective cogito” corresponds to a manifestation of consciousness, because by knowing we are aware of knowing. In order for an individual to be able to reflect, there must be a previous condition of unity with the experience, that is, a pre-reflective presence of the self with itself in the experience. In this way, the pre-reflective “cogito” founds the origins of reflection [10,12,19,20,21].

Pre-reflective consciousness is that self-knowledge in which the subject is always immersed, since it is an experiential form in first person that does not imply the objectification of consciousness. In this way, in a stage prior to reflection, the use of language or propositional meanings is not necessarily required, since pre-reflection is even preliminary to narrative conceptions. The present subjective experience arises from the embodied experience since through the body human beings can become aware of themselves and their interaction with the environment [22,23].

A clear example of these phenomena corresponds to temporality. There is a pre-reflective knowledge of the experience of time that accounts for the experience as such. This means that we live in a chronological and sequentially fluid current of which we are aware without the need to constantly reflect on it. This gives the possibility of reflexively analysing the experience of time from a structural perspective, that is, elaborating questions about time as social construction, analysing the flow of temporal consciousness and reflecting on recognition of past, present and future, among others [24].

Zahavi [12] argues that in Husserlian proposals there are no analyses dedicated exclusively to self-awareness, since they are usually integrated into the nature of intentionality, corporeality, temporality and attention. In this way, it is constituted as a challenge by having to recompose relevant elements to access a deep and complex theory of self-awareness.

First, he criticizes the model of reflection, where it is stated that self-awareness could only occur when consciousness is reflexively objectified. However, he proposes that for reflection to emerge, the subject needs to be part of a whole with the experience. The argument is based on avoiding theories that describe self-awareness as a type of subject–object relationship with experience, constituting the main conflict as it is not perceived from an intimate link between experiential phenomena [12].

From this, he conceives self-awareness as an essential feature of subjectivity and reflection as a grounded and non-basic form of self-awareness. Thus, being a subject corresponds to existing by itself and being aware of itself, since it does not correspond to being something that only occurs in exceptional circumstances, that is, when attention is paid to conscious life, self-awareness is a trait that characterizes subjectivity as such [12].

Gallagher and Zahavi [10] comprehend the dimension of pre-reflective consciousness as an experiential self that is present in every experience in which there is a conscious subject. In this way, they describe it as an invariant dimension in the first person through the multitude of changing experiences. This means that the self preserves its identity while the experiences perish in the stream of consciousness, thus, the self transcends and differentiates itself from the experience but cannot be completely separated from it. In this way, it is constituted as a transcendental self that is experienced pre-reflectively by the individual in each experience or conscious act [12,23].

For their part, Gallagher and Zahavi [10] state that when the body is in immediate and experiential harmony with its environment, it gives rise to a phenomenon known as awareness, where embodied consciousness takes on a fundamental role. In this way, they conceive the body as something beyond an intentional content, since the body allows the first pre-reflective contact with the world and is a determinant of our daily behaviour. Gallagher [25] mentions that by establishing an attention to the body, we can identify it and be reflexively aware of it, considering the body as a mere intentional object. However, bodily awareness cannot be reduced to perceptual awareness. From the perspective that the body is the channel of immediate and pre-reflective contact of the individual with the environment, a proprioceptive and non-perceptual awareness is realized. The present phenomenon refers to an embodied consciousness, not as an object of perception, but rather as an acting and transcendent organism [26].

Awareness in its pre-reflective state is responsible for body awareness in its very essence, allowing us to understand and process the world from immediate experience before more complex cognitive states. In this way, it allows us to develop adaptively in daily actions without requiring a specific intention or attention, for example, walking without tripping, getting dressed or driving a car, achieving a habitual awareness that recreates a passive practice [26].

From the phenomenological postulates, it is possible to state that having consciousness does not necessarily imply an attentional, propositional or cognitive-perceptual process, but rather the possibility of carrying out various human and social practices from a pre-reflective state. This bodily and non-perceptual awareness originates in a background of nature, where the body appropriates the movements, postures and attitudes necessary to respond to the world and its stimuli without generating an explicit or intentional awareness [10,20,26].

Werner [27,28] refers to a similar idea, where he points out that the understanding of the world arises through a bodily perspective, understanding the experience from a holistic way and its relationship with the individual from a phenomenological position. In this way, a relationship first occurs between the organism and its immediate context that gives rise to a series of affective and bodily processes. This phenomenological experience corresponds to a pre-verbal expression known as physiognomic–organismic perception that opens the way, later, for the creation of a propositional meaning, that is, a conceptual scheme regarding the experience [24,27,28,29].

Werner and Kaplan [29] carried out an experiment in which the participants had to observe the fictitious words "budraf" and "medref" to later describe their experience in relation to the word. These words were understood differently and “medref” was even identified as a heavier word than “budraf”. From this, a physiognomic experience is realized before the sound of words, that is, it is possible to grant a meaning through the representation of a sound in relation to the experience itself. Thus, the idea of a preverbal organismic experience that takes place in a stage prior to language and propositional conceptions could be supported [30].

In another study, Werner [28] experimented with subjects who had a language disorder, particularly aphasia. The study consisted of presenting combinations of words and short phrases in a tachyscope so that after a certain time, each participant would refer to their experience in the face of the stimulus presented. Varied reflections emerged that demonstrated the construction of meaning through a bodily physiognomic experience towards the word and its representation before specific recognition. From this perspective, the physiognomization of language manifests an expressive and corporeal nature, allowing certain words to generate sensations from their own experiences even when they are not endowed with a precise meaning, since this allows words such as "stone" to generate a sensation of heaviness [30].

In 1920, the German psychologist Kohler carried out the “Takete–Maluma Effect” experiment which complemented the present postulates by means of a revolution in the language paradigm. Kohler’s study was repeated by Ramachandran and Hubbard, who developed the “Kiki–Bouba Effect”. The study consisted of showing the participants two abstract figures and two meaningless words, “kiki” and “bouba”, with the aim of choosing which word was most identified with the figure, that is, which name was attributed to each. The results showed that 98% of the participants associated the pointed figure with the word “kiki” and the rounded figure with the word “bouba”, indicating the existence of a sensory bias. From this, it is concluded that the symbolic, graphic and sound elements are not subject to a separation between themselves. In this way, an immediate and pre-reflective sensory experience is realized that influences the subject in terms of their sensations and subsequent reflective choice [31].

From these experiments it has been proposed that every state of consciousness implicitly maintains a primitive and pre-reflective consciousness. The present approaches seek to account for the subjective nature of mental states from a phenomenological perspective. From this, it is stated that the object of a conscious and perceptual experience is intersubjectively accessible, since any person can concretely access it. However, the perceptual experience is individual and cannot be shared in its essence with others. In this way, the pre-reflective states related to the same experience are those that make an experience subjective in its origin. Thus, when an experience exists, it is experienced immediately in the first person and initially non-reflectively, generating states that are not transferable to others [32].

## 4. Phenomenology of Intuition

Phenomenology was founded by Edmund Husserl as a response to the prevailing scientific positivism at the end of the 19th century and the beginning of the 20th century, since the premise of a world governed by exact laws that explain, predict and control existing phenomena through generalizations became an extremely reductionist and limiting scenario. In this way, phenomenology proposes a new form of relationship with the environment, where it is sought to know the phenomena in their essence and purest state, that is, without manipulating the object of study to truly know it [33,34].

Husserl [7,8] states that phenomena are what is presented in consciousness, since it consists of an appearance through immediate perception. From this, one of the fundamental premises of phenomenology arises, which proposes that phenomena are made present in their essential content through intuition, that is, a way of access to the knowledge of the objects of the world in a direct way, not mediated by reason. Subsequently, in the subjective experience a reflexive, propositional and referential process emerges [7,8].

In this way, it is proposed that every experience that reaches a reflective perspective implicitly entails an essence that provides depth to the experience, that is, a content that can be perceived intuitively and experienced in its essential origin [8].

Husserl [8] defines ideational abstraction or intuition of essences as a human faculty that can be regularized methodically to perform a phenomenological analysis of consciousness. For this, he posits that intuition is based on the immediate experimentation of individual objects, that is, in the possibility of focusing on the object or representation from its essential perspective. In this way, it is essential to generate a parenthesis in the singular or relative existence to access the purest of the experience. The intuition of essences or the eidetic method intends not to be limited to exclusive empirical facts, since it seeks to capture the prior interactions and that which remains invariant in all the possible singular cases of the object, that is, the essence. This being so, the phenomenological intuition would correspond to the rationality of reason, that is, the principle of all principles that is not empirically evaluable [34,35].

From this perspective then, the objects of immediate perception are captured directly, abruptly and intuitively, since they are available to be the object of an intention. Intentionality is a fundamental characteristic of consciousness and intuition of essences from the phenomenological perspective. In this way, consciousness and intuition are always directed towards something, that is, towards an object of which we are aware [8,35].

On the other hand, in Husserl's work the intuition of essences is differentiated from the categorical intuition. The latter requires a succession of articulated acts, a new intention and higher level processes. In this way, it does not correspond to a phenomenological intuitive act but to a founded, categorized and intentional cognitive act [35]. However, according to Husserl [9] in *Logical Investigations*, there is a close link between categorical intuition and essence or eidetic intuition, since it arises from the distinction between synthetic and abstract categorical acts. Synthetic acts are constituted as unifying and capture the object from a relational perspective, and abstract acts allow us to reach pure meaning through abstraction. In this way, through abstractive categorical acts, it is possible to address the essence of things [9,36].

Bergson [37], for his part, argues that philosophers require preliminary foundations and old theories to generate new connections, since they are interconnected and evolve over time. However, there is a fundamental simplicity within the doctrines that is known as intuition. Every approach arises from an initial intuition that the philosopher tirelessly tries to put into words without success, which is why he tends to correct and elaborate again his proposals with the aim of getting closer and closer to the original intuition. In this way, the doctrines are an incommensurability of the simple intuition and the means that the subject has to express it; thus, people manage to elaborate an image of the original intuition of a formula through the abstractions that are used to manifest it [37].

All the comings and goings of the philosophical proposals are attempts to express their intuition, falling mostly into symbolic approaches, explanatory and conceptual theories that distance themselves from the fundamental essence. It is not possible to identify as a constitutive element that which was exclusively the means of expression, since the doctrines change over time, being more an intuitive evolution than a mere composition [37].

Philosophy does not arise from pre-existing ideas, since it emerges from an intuition that is transformed into ideas or abstract elaborations that allow us to try to express its primary essence. In this way, the word is constituted as a reduced element of the sense and initial intuitive movement. Thus, through conceptual constructions, limited in their exclusivity, an opening is generated to approach the primary intuition, giving rise to a whirlwind of impulses that favour an approach to the essence [37].

For Bergson [37], the only way not to reduce the philosophical attitude is through intuition. This is not a mere form of immediate access but corresponds to a metaphysical approach to existence, since it constitutes an act that generates contact with the being and in which all vital forces are involved. In this way, it prioritizes action, understood as an encounter between life and matter [38].

Bergson [37] states that the intellectual attitude is based on science and the empirical, where there are static objects that can be decomposed and composed successively. In this way, it is constituted as a mechanical reality and the superficial aspect of existence, because under it a deeper and more authentic reality characterized by intuition manifests itself. In the present reality there is a constant movement and a flow of time, where the person can immerse himself in a complete experience to later generate reflexive or verbal elaborations [39].

This means that when we perceive an object, two possibilities of movement are generated: relative and absolute. From the relative position, there is a different perception depending on the point of view, since the symbols, interpretations and subjectivity are in interaction when they meet from the outside of the object. This operation corresponds to an analysis that reduces the object to those elements already known to provide an order and structure. The multiplicity of translations of something can add extremely useful nuances in terms of understanding the object from different visions; however, they do not allow us to know the essence of the object. This is because it is not possible to understand the essence of things from a spectator perspective [37].

For its part, an absolute movement implies entering the interior of the object, that is, its original essence or state of the soul. In this way, it is constituted as a perfect state by being perfectly what it is without relativism. From this, it follows that the absolute arises through intuition, being a way to transport oneself to the interior of the object in order to know its authenticity and inexpressible character [37].

Bergson [37] proposes the importance of reaching the absolute to know the objects of the world in their essence, for which it values intuition over analysis as a method to achieve its objectives. In this way, he proposes that metaphysics is the science that intends to refrain from symbols, that is, those representations of the interior to focus directly on interiority from a phenomenological perspective.

From this perspective, Bergson [37] postulates the infeasibility of experiencing conscious life first through reflection and cognitions, since these correspond to constructions of the intellect that stop the duration and freedom of spiritual life [38]. The duration corresponds to the reality of consciousness without a structure of the intellect, since it refers to the flow of consciousness and time in a continuous way. In this way, memory and future experiences are a succession of spiritual states that arise without interruption [40].

One of the main examples regarding the ability to capture a reality from within corresponds to the intuition of duration, since it corresponds to the possibility of perceiving the flow of time and the person himself. The analytical–reflexive dimension of consciousness operates from the immobile, concrete, invariable and static, since it is a symbolic reconstruction and a simplified scheme that remains and does not vary in duration. For its part, intuition operates from mobility and variability in the fluidity of time, that is, in duration. This is because the phenomenological and intuitive experience lies in a succession of sensations of the integral experience [37].

According to Bergson [37], scientists and psychologists have categorized and fragmented human experience so that psychological states are manageable, undoubtedly a necessary measure for analysis and effective therapeutic approach. However, this simplification of the person reduces fundamental aspects of his interiority, since it overlooks the nuances that are inexpressible and unique, that is, intuitive. Thus, the real and internal organization of an object is transformed into an external and schematic reconstitution through the choice of a mode of representation and a specific point of view. It should be noted that it is possible to fragment a primary intuition through a detailed analysis; however, it is not possible to carry out the reverse process. This means that we cannot arrive at an unknown primary intuition through the perception of the schematized fragments, namely, the reflexivity of consciousness. For example, a person who goes to Paris and is immersed in an immediate and intuitive experience can fragment it representationally through a drawing of Parisian landscapes and even reconnect with that intuitive experience by visualizing it. However, a person who has not been to Paris and has not experienced its streets phenomenologically will not be able to arrive at an intuition originating in Paris by visualizing a symbolic fragment [37].

It is essential to point out that the intuitive method proposed by Bergson [37] corresponds to a method and not a capacity of the mind. In this way, it is not constituted as a magical internal experience but a new type of empiricism that integrates a pragmatic commitment with the world. He proposes a method in which metaphysics and science converge with the aim of being able to expand the limits of study and not focus exclusively on the static, that is, the measurable. From this perspective, it is argued that science and human physiological nature fail to capture the aspects of life in relation to time and movement, since it remains immutable and perceives variation as an aspect that escapes scientific control by what could not seize the true movement [41].

Notwithstanding the foregoing, there are some nuances that differentiate the notion of intuition in the work of Husserl [11] and Bergson [37]. In Husserl, for example, intuition is articulated mainly through conceptual elements (both as categorical intuition and as eidetic vision, or in a less elaborate, but still articulated way, in the case of pre-predicative experience). In Bergson, on the other hand, intuition is something inexpressible, something unique, something ineffable, which is only experienced and not accessed categorically.

In Husserl [11], in one way or another, we can always articulate and express what is given or implied in the immediate intuition, while in Bergson [37], the intuitive experience is lived but not expressed.

The present critique of the assumption of a rigid intellect, adapted to pre-existing molds and with a growing overvaluation of universal mathematics, favours the creation of a new empiricism. In this way, through a method that allows us to understand the evolution of being and capture the mobile aspects of experience through intuition, the flow of duration could be addressed and not exclusively the action itself [41].

For his part, Levinas posits that existence begins with the phenomenological intuition of being and understands it as the experience of duration and of the spiritual, being irreducible to mere knowledge. On the contrary, intuition would be responsible for promoting thought and knowledge. In this way, he conceives that the truth of thought in relation to an intentional object originates in intuition, since it is constituted as an original phenomenon that makes truth itself possible [38,42].

Levinas reinforces intuition as a fundamental piece of existence and as a means to reach fullness, since it corresponds to a mode of consciousness through which one enters into full contact with existence. In this way, he seeks to detect the original place and understand its essential characteristics to subsequently give rise to a reflective space, since he considers reflection to be the last level of the intuitive process [38,42].

## 5. An Intuitive Pre-Reflective Way to Approach Others

Based on the above, it is possible to think of the existence of a pre-reflective intuitive dimension in the encounter with another. This dimension is at the base of ToM and reflexivity. There is an understanding in psychology related to the fact that to know ourselves and to know others we need a theory about our own subjective states and, therefore, only in this way can we obtain a subjective theory of the other. However, ToM, widely studied in psychology, does not incorporate the most basic and primitive dimensions of the functioning of consciousness, namely, the intuitive and pre-reflective dimension of human experience. These two forms, pre-reflective intuitive and reflexive rational (ToM), are not mutually exclusive. In the encounter with others, both dimensions of consciousness are displayed and dialogue with each other. The apprehension of the other is always a pre-reflective intuitive experience rather than a reflective rational experience. The other has a direct impact and it is only possible to build a theory taking intuitively acquired knowledge as input. By this we mean that in intersubjective experiences there is a form of knowledge about the other and oneself that is not cognitive-intellectual knowledge, but rather tacit, non-propositional and non-declarative knowledge [43]. The intuitive direct experience of the other is a form of knowledge in itself and does not require a mental re-presentation of the other's subjectivity. That theory of which ToM speaks is a phenomenal theory, it is an experience of the other, which is constructed and co-constructed at each moment of the relationship and likewise varies over time, is modified and transformed. The other is presented to consciousness in a direct, intuitive and pre-reflective way and it is only possible to build a “theory” of his mental states through reflection and executive control of psychological functions.

From the perspective developed in this article, ToM has received a strong cognitive influence, probably due to the decades and century in which it emerged. Psychology has been governed primarily by the cognitive sciences for much of the 20th century, abandoning other traditions and theories that may strengthen understanding of psychological phenomena. ToM has produced a mechanization of intersubjectivity, influenced by the computational model of the mind, thus eliminating the world of life [6,7,9].

The pre-reflective intuitive experience involved in the process of understanding the subjectivity of the other is a first-person experiential form that does not imply the objectification of consciousness [22], since, as stated by García [23], in a pre-reflection stage the use of language or propositional meanings is not necessarily required since pre-reflection is even preliminary to narrative conceptions.

From Bergson’s [37] perspective, ToM is an attempt to express intuition using symbolic approaches, explanatory and conceptual theories that distance themselves from the fundamental essence. This is because there are ways of knowing that are not reflective, but intuitive, pre-reflexive and pre-verbal. The word is limited in its use to understand one's own human experience and that of others. Following Bergson [37], one must rely more on the pre-reflective and pre-verbal dimension of experience. The word is constituted as a reduced element of sense and intuitive knowledge. However, through conceptual constructions, limited in their exclusivity, an opening is generated to approach primary intuition. The word, in its essence, constitutes only a vehicle for channelling tacit, intuitive and pre-reflective knowledge [44].

When Bergson [37] proposes the existence of two dimensions in experience, one in which there are static and mechanical objects that can be decomposed and that, on the other hand, there is a deeper and more authentic dimension characterized by intuition, he refers that the reflexive–intellectual aspects and the pre-reflective intuitive aspects are permanently intertwined in the flow of human experience. This relates directly to the two forms of human perception described by Werner [27,28]; namely, the geometric–technical dimension and the physiognomic–organismic dimension. According to García [39], it is possible to think of the existence of an intuitive flow of time where the lived experience takes place to later generate reflexive elaborations dominated by the word.

In this sense, the reflexive and the pre-reflective constitute a complex unit. The intuitive approach to the experience of the other occurs at the same time as the emergence of reflexivity. In this sense, a dynamic, dialogical and dialectical relationship is established between the reflective dimension of the experience and the intuitive pre-reflective dimension in the encounter with the other. When Gallagher and Zahavi [10] state that the body and embodied consciousness have a fundamental role in awareness, they mean that the body is the main route for pre-reflective intuitive contact with others and the world. This means that there is a direct relationship between pre-reflexivity and pre-verbal experience, as well as a direct relationship between pre-reflexivity and the body [27,28,29].

Theodor Lipps [45], in the beginnings of psychology, proposed the notion of *Einfühlung.* Although Lipps [45] takes this concept of the aesthetic impact that art generates in the human experience, later it is extrapolated to intersubjective interactions, accounting for a moment-by-moment cognitive–affective connection and synchrony that enables the co-construction of the intersubjective process of mutual understanding and empathy. Following Lipps’ approach [45] it is possible to think that the notion of ToM lacks the notion of emergence, novelty and temporality. A “theory” is not necessary to understand the other; we need to be WITH the other. Rather, what ToM proposes is closer to the notion of anticipatory pre-memory in the face of the encounter with the other, widely developed in the work of Husserl [11], which constitutes a lived memory, not conceptual and non-representational.

However, the notion of empathy that Husserl [46] develops in “The Phenomenology of Intersubjectivity” differs in one aspect from the notion of empathy developed by Lipps [45]. For Husserl [46] empathy is not a duplication of the self in another object. The other, from the Husserlian perspective, is captured directly as an intuitive experience. The other is not a projection of myself; the other is not a projection of internal states onto an external object, as Lipps [45] points out. While one understands empathy as a perceptual experience, the other understands it as an apperceptive experience.

What we have developed in this work is that the process of understanding others does not occur through what has been described as ToM, but through a co-phenomenology [47]. This is a coordinated and synchronous intersubjective process, which is not generated in the “cognitive system” of one of the participants, but in the phenomenal emergence of the intuitive and pre-reflective encounter.

## 6. Conclusions

In this work we have tried to expand the notion of ToM with elements of phenomenology, such as the theory of intuition and pre-reflective consciousness. ToM, understood as the ability to intuit one’s own mental states and those of others, has been deeply investigated in developmental psychology and cognitive psychology; however, it has not integrated elements of phenomenological philosophy that could provide greater depth in mutual understanding. Although the psychological literature shows a direct relationship between ToM and the reflective capacity of consciousness, in this article we have established a direct relationship between intuition and pre-reflexivity. Pre-reflective, pre-verbal, non-propositional intuitive experience is completely enmeshed in the process of developing a theory about the subjective states of others.

This article constitutes a contribution to the integration of theoretical approaches in psychology and phenomenology. Future experimental phenomenological border studies between phenomenological philosophy and psychology should delve into how both dimensions of consciousness involved in the process of cognitively and affectively approaching others are manifested empirically.

## Data Availability

This article is not include data.
